# Nutritional and lifestyle changes required for minimizing the recovery period in home quarantined COVID‐19 patients of Punjab, Pakistan

**DOI:** 10.1002/fsn3.2458

**Published:** 2021-07-09

**Authors:** Roshina Rabail, Javeria Saleem, Zunera Tanveer, Simon G. Patching, Abdur Rauf Khalid, Muhammad Tauseef Sultan, Muhammad Faisal Manzoor, Emad Karrar, Muhammad Inam‐Ur‐Raheem, Muhammad Asim Shabbir, Rana Muhammad Aadil

**Affiliations:** ^1^ National Institute of Food Science and Technology University of Agriculture Faisalabad Pakistan; ^2^ Department of Public Health, Institute of Social and Cultural Studies University of the Punjab Lahore Pakistan; ^3^ Institute of Molecular Biology and Biotechnology (IMBB) University of Lahore Lahore Pakistan; ^4^ Department of Physiology Bolan University of Medical and Health Sciences (BUMHS) Quetta Pakistan; ^5^ School of Biomedical Sciences and Astbury Centre for Structural Molecular Biology University of Leeds Leeds UK; ^6^ Department of Livestock and Poultry Production Faculty of Veterinary Sciences Bahauddin Zakariya University Multan Pakistan; ^7^ Institute of Food Science and Nutrition Bahauddin Zakariya University Multan Pakistan; ^8^ School of Food and Biological Engineering Jiangsu University Zhenjiang China; ^9^ Department of Food Engineering and Technology Faculty of Engineering and Technology University Gezira Wad Medani Sudan

**Keywords:** COVID‐19, lifestyle, nutrition, recovery, vitamin supplements

## Abstract

The COVID‐19 pandemic has introduced a new battle in human history for a safe and fearless life. Therefore, this cross‐sectional survey was conducted (Punjab, Pakistan) on healthy recovered, home quarantined COVID‐19 patients to draw conclusive health support guidelines in the fight against this pandemic. COVID‐19 recovered patients (*n* = 80) of age ≥14 years were randomly selected during the period November 2020 to February 2021. A nutrition and lifestyle changes questionnaire, containing ten sections and seventy questions, was completed through the telephone/WhatsApp. Data were transferred into an Excel spreadsheet and statistically analyzed by applying chi‐square, correlation, and a *t* test of independent values using SPSS‐16 software. The patients had an age range of 14 to 80 years, of which 52 (65%) were male and 28 (35%) were female, and 32 (40%) had a normal BMI. The patients had a peak COVID‐19 recovery period of 2 weeks, and a mean recovery period of 2.8 ± 1.4 weeks. Certain variables, including gender (males), age (>40 years), sleep (≤5 hr), less/no physical activity, obesity, diabetes mellitus, and autoimmune diseases, were significantly associated with delayed recovery. Poor nutritional outcomes, including lower intakes of water, legumes, nuts, meat, and milk/yogurt; and higher consumption of fast/fried/junk/spicy foods and cold water/drinks, were also significantly associated with a longer recovery period. The results were similar for not taking daily doses of multivitamins, and vitamins C, D, E, and zinc. This study identified that staying physically active, maintaining sensible body weight, having a sleep of 7 hr, consuming more foods of plant origin especially plant‐based proteins from nuts and legumes, taking supplemental doses of multivitamins, vitamin D, E, and zinc, along with drinking ≥2 L of water daily can provide a significant role in early and safe recovery from COVID‐19.

## Graphical abstract

Timely nutritional and lifestyle changes can help save us and our future generations. Based on this study, we have concluded many beneficial nutritional guidelines by examining the nutritional patterns adopted by COVID‐19 recovered home quarantined patients. A healthy lifestyle with appropriate sleep hours, steam inhalation, and physical activity could help fight COVID‐19 infection more effectively and/or more quickly.

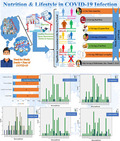

## 1 INTRODUCTION

The World Health Organization (WHO) was notified about abundantly rising unknown pneumonia cases in the city of Wuhan, China, during December 2019 that were later announced like a novel coronavirus (COVID‐19). This outbreak brought large‐scale human threat and was defined as a pandemic by the WHO (Messina et al., 2020; Naja & Hamadeh, 2020). The threats associated with this coronavirus are possibly due to the uncontrolled production of pro‐inflammatory cytokines. It is similar to other highly virulent respiratory viruses, which result in significant numbers of critical care patients in intensive care units (9%–11%) and a significant mortality rate (5%–7%). Hence, appropriate steps to control and treat this viral infection had to be taken (Messina et al., 2020).

The current COVID‐19 pandemic has directed the focus of nutrition research from noncommunicable diseases toward communicable disease. The public, researchers, and healthcare professionals are generally unaware of how diet influences COVID‐19, but consumption of a well‐balanced diet to encourage normal B‐ and T‐cell functioning could be helpful (Jaggers et al., 2020). Chronic pathologies in COVID‐19 patients (Brugliera et al., 2020) may decrease the micronutrient status of the body, hence increasing their demands from recommended dietary allowances (Thibault et al., 2020), so eating a balanced diet and maintaining a healthy lifestyle is vital (FAO, 2020). Diet is not a cure for COVID‐19, but it is a modifiable factor in its development (Kamyari et al., 2021) that can help minimize infection progression and enhance recovery (Aman & Masood, 2020). Specific nutrients can influence the immune system by activating cells, altering signals and gene expression, and determining gut microbial composition. Among those important micronutrients are zinc, vitamins A, D, E, B6, B12, and C (Jaggers et al., 2020; Naja & Hamadeh, 2020), and the macronutrients are proteins and polyunsaturated fatty acids (Messina et al., 2020; Thibault et al., 2020). Certain other non‐nutritional food constituents have also been reported productive for immune system modulation, such as polyphenols and flavonoids (Manzoor et al., 2017, 2019; Messina et al., 2020). People with a healthy immune system will battle COVID‐19 in a better way (Nizami & Uddin, 2020).

In around 80% of COVID‐19 infected patients, the disease will be mild to moderate, confined to the upper respiratory tract, and can be managed at home with proper care and conservative symptomatic therapy (Chowdhury et al., 2020). The individual responsibility of the whole of human relies on making an effort to live a balanced lifestyle, consume a diet rich in fruits and vegetables, exercise regularly, maintain a healthy weight, and get enough sleep (Naja & Hamadeh, 2020). Hence, this whole scenario has generated a need to learn successful outcomes from those who have recovered with a minimum recovery period and with minimum pathophysiology. Therefore, this cross‐sectional survey‐based study was conducted to recognize successful nutritional and lifestyle changes adopted by COVID‐19 patients for rapid recovery during home quarantine in Punjab, Pakistan.

## 2 MATERIALS AND METHODS

### 2.1 Survey methodology

The survey was conducted from November 2020 to February 2021. Patient inclusion criteria for the study were that they had completely recovered from COVID‐19 infection at home. Patients with a confirmed case of COVID‐19 detected by recommended methods (Hariri & Narin, 2020; NIH, 2020) such as reverse transcription‐polymerase chain reaction (RT‐PCR), chest computerized tomography (CT) scans, and serological enzyme‐linked immunosorbent assay (ELISA) for IgG/IgM antibodies were given preference, but a few cases with a physician's strong suspicions were also included. In the period of the study, a total of eighty (*n* = 80) COVID‐19 healthy recovered, home isolated, Pakistani nationals/residents from different cities of Punjab were contacted by phone to complete the questionnaire (collection of data) and to confirm reports of their COVID‐19 diagnosis. All participating patients were preinformed and guided about the study objectives, including that the given information was only required for positive outcomes and only selected outcomes were to be shared according to the privacy policy, and their identity and contacts will not be disclosed at any point. A purposive selection of COVID‐19 healthy recovered (age ≥14 years) patients was done without discriminating gender or clinical, nutritional, and socio‐economical background for reducing biasness and enrichment of data.

### 2.2 Development of a questionnaire for COVID‐19 recovered patients

A questionnaire was developed by making some appropriate changes and additions after pilot testing of a questionnaire conducted by Di Renzo et al. (2020). The questionnaire was composed of seventy questions on the following ten categories: (I) personal data (four questions on age, gender, occupation, hometown); (II) anthropometrics (four questions on height, weight, BMI, body weight change); (III) COVID‐19 updates (four questions relevant to infection and detection); (IV) apparent symptoms (fourteen questions); (V) past medical history (four questions on lung disease (bronchitis/asthma), autoimmune disorders (arthritis/allergies/hypersensitivities), chronic diseases (cardiovascular/hypertension/renal), and diabetes mellitus); (VI) nutritional changes (twenty two questions on dietary consumption patterns); (VII) nutritional supplements and drugs (seven questions on taking multivitamins and/or individual nutrients, and medications including analgesics, antipyretics, and antibiotics); (VIII) lifestyle changes (six questions on smoking, sleeping hours (≤5, 6, 7, 8, 9≥), physical activity (very active, fairly active, less/not active), steam inhalation, blood pressure, blood glucose, oxygen level monitoring); (IX) recovery time (two questions on days quarantined, days for complete recovery) and (X) post‐traumatic stress disorder (PSTD) (three questions on stress/anxiety, fears, PSTD). The questionnaire has been attached in the Appendix 1. The questionnaires were completed by telephone call, and WhatsApp was used to collect reports of COVID‐19 diagnosis. Once completed, survey answers were transferred to a Microsoft Excel spreadsheet.

### 2.3 Statistical analyses

Numerical data were tabulated in Excel spreadsheets and analyzed using SPSS‐16 software (IBM). Associations and correlations were calculated among different variables. Statistics from the following analyses were tabulated and used to interpret the results: Chi‐squared (χ^2^), likelihood ratio (LR), linear by linear association (LA), Lambda (λ), Goodman and Kruskal tau (GK), gamma (Γ), Spearman's correlation (r_s_), Pearson's R (r_p_), and frequency distribution. An independent sample *t* test was applied for comparison. The significance level was set as *p* < .05 (*p ≤ .05; **p ≤ .01; ***p ≤ .001).

## 3 RESULTS

### 3.1 COVID‐19 infection, detection, and recovery

**FIGURE 1:**
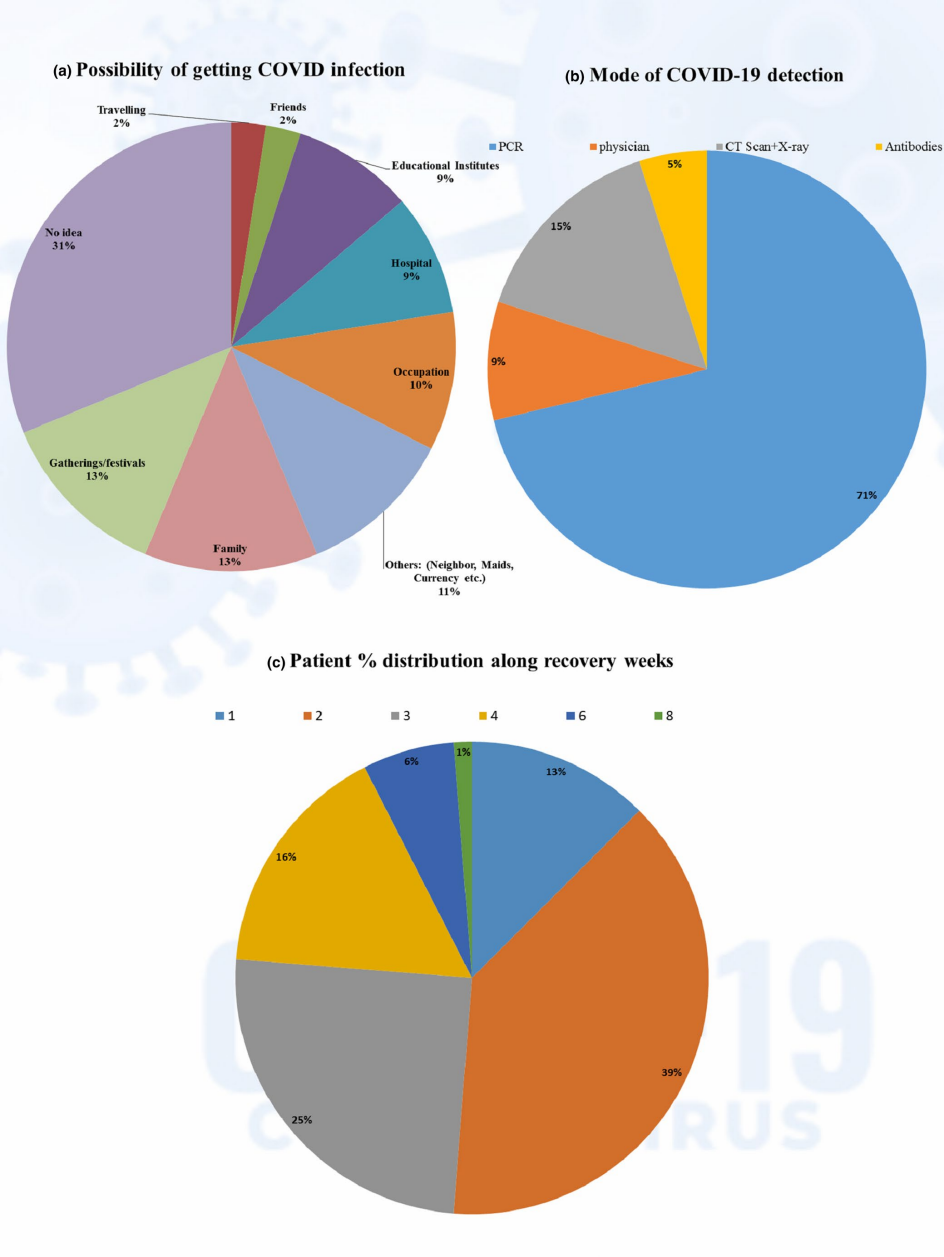
Outcomes (%) for COVID‐19 infection, detection and recovery period

It is clear from Figure 1a that most patients had no idea from where they became infected with COVID‐19. On the other hand, gatherings like wedding ceremonies, religious festival (Eid), funerals, market places, and family members were among major infection contributors. Places of work, hospitals, and educational establishments were also of significance for transmitting the virus. The modes for COVID‐19 detection vary widely, but the criteria for mode selection depend upon the economical feasibility of patients and physician's preferences. The mode most adopted for detection was RT‐PCR as shown in Figure 1b. The IgG/IgM antibodies test was least used for detection on its own; rather it was used in combination with other detection modes for confirmation purposes. Results on COVID‐19 exposure revealed that 44.8% of patients had COVID‐19 infected members in their family during the past month, while 37.5% had a travel history to an infected area. The most common recovery time was reported as 2 weeks followed by 3 weeks as shown in Figure 1c, while some recovery was also reported in the first week. The overall mean recovery period was 2.8 ± 1.4 weeks or 19.2 ± 10.5 days.

### 3.2 Age, gender, BMI, and body weight change in COVID‐19

**FIGURE 2:**
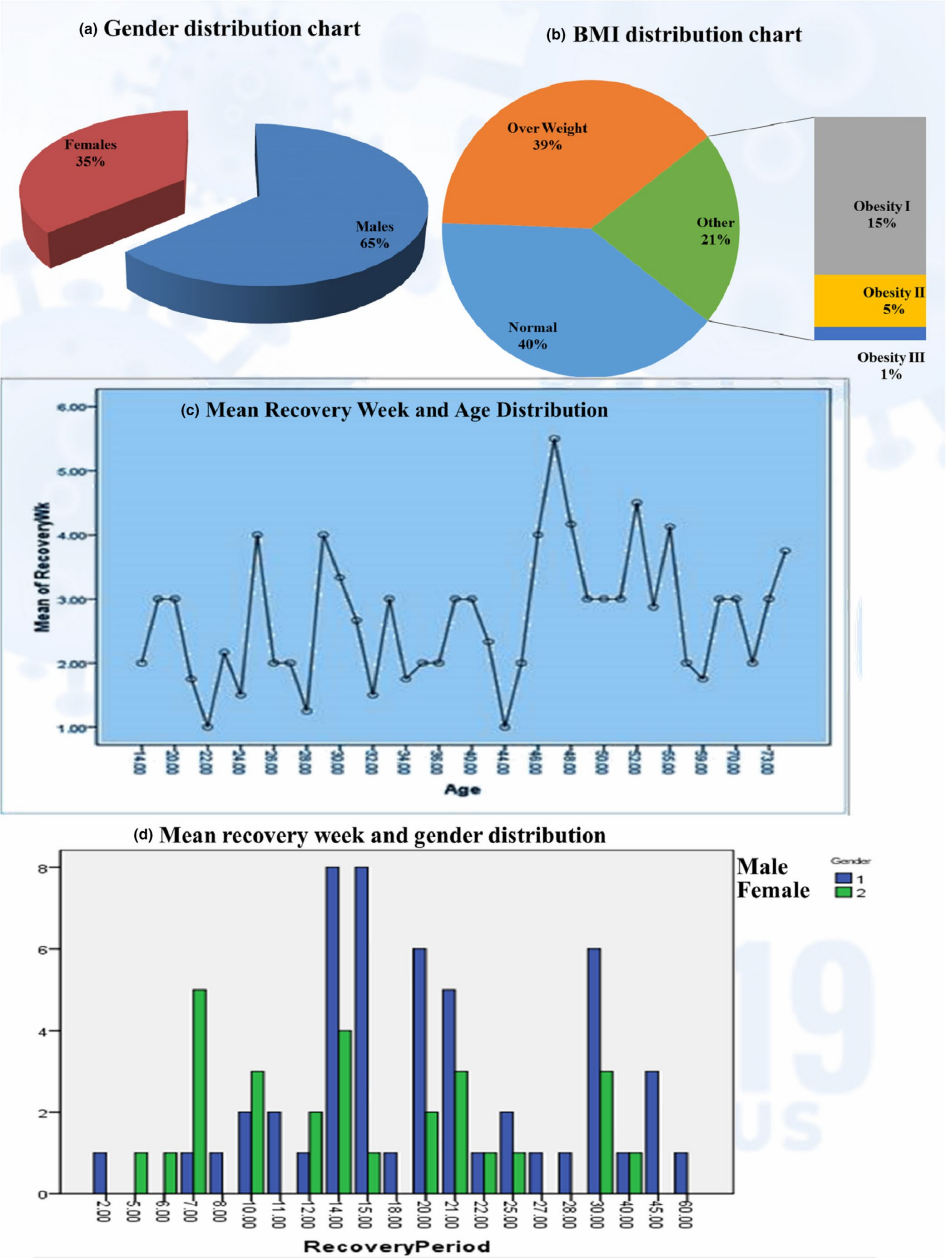
Age, gender, BMI distribution

Table 1 shows the descriptive statistics of patients' age, gender, BMI, and recovery period. The percentage distribution for gender as shown in Figure 2a reveals that males were almost double in numbers compared to the females. Statistical analysis revealed significant associations and correlations between gender and recovery (LA =3.811, *p* = .051^*^; λ = 0.286, *p* = .040^*^; Γ = −0.315, *p* = .032^*^; rs = −0.243, *p* = .030^*^; r_p_ = −0.220, *p* = .050^*^). Here, negative values of symmetric measures mean an inverse relation indicating an increase in recovery period with an increased number of males. The results of the *t* test also support a difference in variances between male and female patients for recovery (t = 1.988, *p* = .050^*^), and similarly, data from Figure 2d also demonstrate more male patients with fourteen or more days of recovery. The age distribution in Figure 2c reveals that recovery longer than 3 weeks was more associated with increased age. Among different age groups, the highest frequencies for COVID infection were reported for students aged 23 years and professionals aged 34 years. The statistical comparison of age versus recovery time revealed significant associations and correlations (LA =6.925, *p* = .008^***^; λ = 0.284, *p* = .000^***^; Γ = 0.223, *p* = .001^***^; r_s_ = 0.310, *p* = .005^***^; r_p_ = 0.296, *p* = .008^***^), indicating an ordinal slight positive increase in recovery time with increasing age. To analyze the differences between age groups at 40 (<40 and ≥40), a *t* test was applied, for which highly significant results were obtained (t = 3.074, *p* = .003^***^).

**TABLE 1:** Descriptive statistics of age, gender, BMI, and recovery

	*N*	Range	Min	Max	Mean	*SEM*	*SD*
Age	80	66	14	80	40.4	1.711521	15.30831
Gender	80	1	1	2	1.35	0.053663	0.479979
BMI	80	21.4	18.6	40	27.08	0.549353	4.913567
Recovery (Days)	80	58	2	60	19.2375	1.176678	10.52453
Recovery (Weeks)	80	7	1	8	2.775	0.154033	1.377716

Abbreviations: *N*, Number of patients; *SD*, standard deviation; *SEM*, standard error of mean.

BMI was calculated from the given data on height and weight by applying the Quetelet equation (body weight in kg/height in m^2^) and categorized according to WHO criteria (Pi‐Sunyer, 2000) into the following five groups: (a) normal weight (18.5–24.99 kg/m^2^), (b) overweight (25.0–29.99 kg/m^2^), (c) obesity grade‐I (30.0–34.99 kg/m^2^), (d) obesity grade‐II (35–39.99 kg/m^2^), and (e) obesity Grade‐III (>40 kg/m^2^) (Ashwell & Gibson, 2016). The patients had a mean BMI of 27.1 ± 0.5 and the BMI distribution revealed almost equal proportions for normal weight and overweight patients, while obese patients had half of this proportion (Figure 2b). BMI was shown to have a significant dependency on recovery time (*χ*2 = 1.275, *p* = .027^*^; λ = 0.219, *p* = .000^***^). Bodyweight changes with COVID‐19 infection were reported as gain (20%), loss (35%), and stability (45%) and were found to significantly affect the recovery period (λ = 0.341, *p* = .005^***^). Patients who reported a loss in body weight were shown to have late recoveries as indicated in Figure 2b, Table 2.

**TABLE 2:** Frequency and percentage distribution of complete study data

	Variables		Frequency	Percent%
Personal data	Gender	Male	52	65
		Female	28	35
	Age	<40 years	40	50
		≥40 years	40	50
	BMI categories	Normal	32	40
		Overweight	31	38.8
		Obesity I	12	15
		Obesity I	4	5
		Obesity III	1	1.2
COVID−19	Recovery week	14 days	32	40
		28 days	33	41.25
		42 days	11	13.75
		>42 days	4	5
	COVID−19 detection	rt‐PCR	57	71.2
		CT‐Scan	13	16.2
		Antibodies	3	3.8
		Physician's suspection	7	8.8
Drugs & supplements	Nutritional supplements	Multivitamin	33	41.2
		Vitamin A	3	3.8
		Vitamin D	19	23.8
		Vitamin C	34	42.5
		Vitamin E	4	5
		Zinc	24	30
	Drugs	Analgesics& antipyretics	52	65
		Antibiotics	38	47.5
Major food consumption pattern	Water	<2 L	48	60
		≥2 L	32	40
	Egg	<4	25	31.2
		≥4	55	68.75
	Nuts 30 gram/week		54	67.5
	Fish		24	30
	Meat 300−400 g/week		47	58.8
	Legumes ≥3 serving/Week		52	65
	Fruit ≥3 servings/Day		59	73.8
	Vegetable ≥2 servings/Day		60	75
	Fat/Oil <5 Tbsp./Day		51	63.8
	Onion/Garlic/Tomato		51	63.8
	Carbonated drinks <1 serving/Week		31	38.8
	Sugar/Pastries/desserts <3 servings/Week		12	15
	Pasta/Rice ≤1 (80 grams) serving/Day		53	66
	Milk/Yogurt ≥2 servings/Day		42	52.5
Nutritional modifications	Meat added		23	28.8
	Broth/Soups added		30	37.5
	Fruits added		66	82.5
	Vegetables added		29	36.2
	Eggs added		17	21.2
	Nuts added		19	23.8
	Citrus added		7	8.8
	Ginger added		14	17.5
	Green tea added		46	57.5
	Senna Makki added		6	7.5
	Rice avoided		12	15
	Cold drinks avoided		26	32.5
	Spice foods avoided		23	28.8
	Junk/Fried/fast foods avoided		45	56.2
Stress	Anxiety/stress		53	66.2
	Fear of death		51	63.8
	PSTD		14	17.5
Weight, diet	Weight change	Gain	16	20
		Loss	28	35
		Stable	36	45
	Diet improved	Improved	57	71.2
		Worsen	23	28.8
Diseases	Lung disease		11	13.8
	Autoimmune disease		4	5
	Chronic disease		8	10
	Diabetes mellitus		11	13.8
Symptoms	Sore throat		59	73.8
	Cough		61	76.2
	Fatigue		74	92.5
	Breath difficulty		55	68.8
	Fever		73	91.2
	Fever consistency		39	48.8
	Fever intensity	High	27	33.8
		Moderate	37	46.2
		Mild	16	20
	Muscular pain		64	81.2
	Loss of senses		65	81.2
	Feeling asleep		40	50
	Chest pain		42	52.5
	Confusion		37	46.2
	Headache		13	16.2
	IBS		8	10
Life style	Smoking		7	8.8
	Sleep hours	<7 hr	30	37.5
		≥7 hr	50	62.5
	Physical activity before COVID−19	Very active	5	6.2
		Fairly active	37	46.2
		Less/No active	38	47.5
	Physical activity during COVID−19	Very active	5	6.2
		Fairly active	41	51
		Less/No active	34	42.5

### 3.3 Past medical history and apparent symptoms in COVID‐19 infection

**FIGURE 3:**
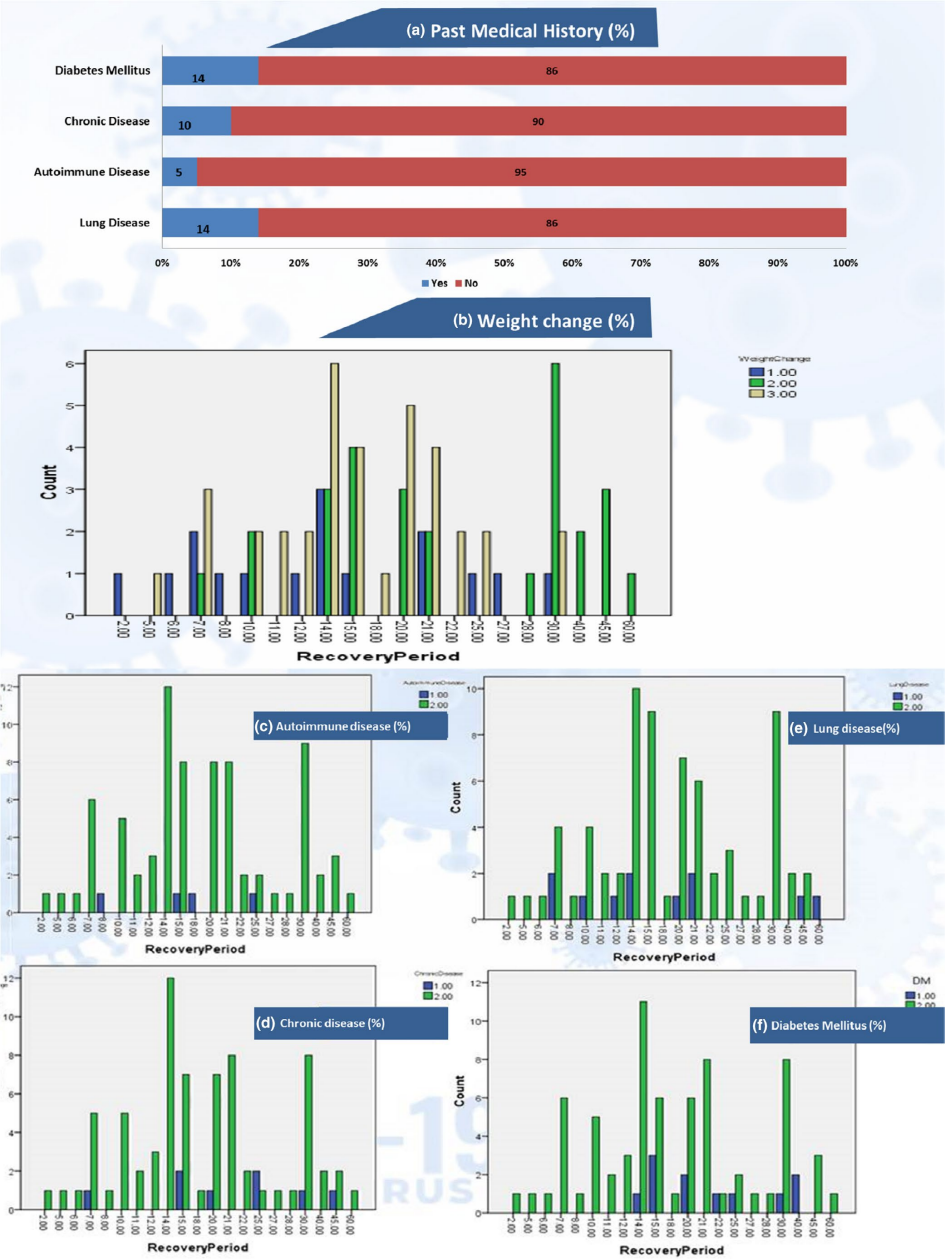
Body wt. changes and past medical history distribution along recovery period. ^*^a. (% disease distribution); b. weight change (1 = gain, 2 = loss, 3 = stable), c. d. e. f (1 = yes, 2 = no)

Results for past medical history are shown in Figure 3a. Surprisingly, there were no significant associations or correlations of lung and chronic disease with a recovery period. But results for autoimmune disorders were highly significant (*χ*2 = 47.251, *p* = .001^***^; GK =0.591, *p* = .001^***^). There was a significant ordinal negative correlation (Γ = −0.361, *p* = .038^*^) between diabetes and recovery, which indicates a slight ordinal decrease in diabetic patients along with an increase in recovery as well as more diabetes mellitus patients clustered around the late recovery zone as shown in Figure 2f.

**FIGURE 4:**
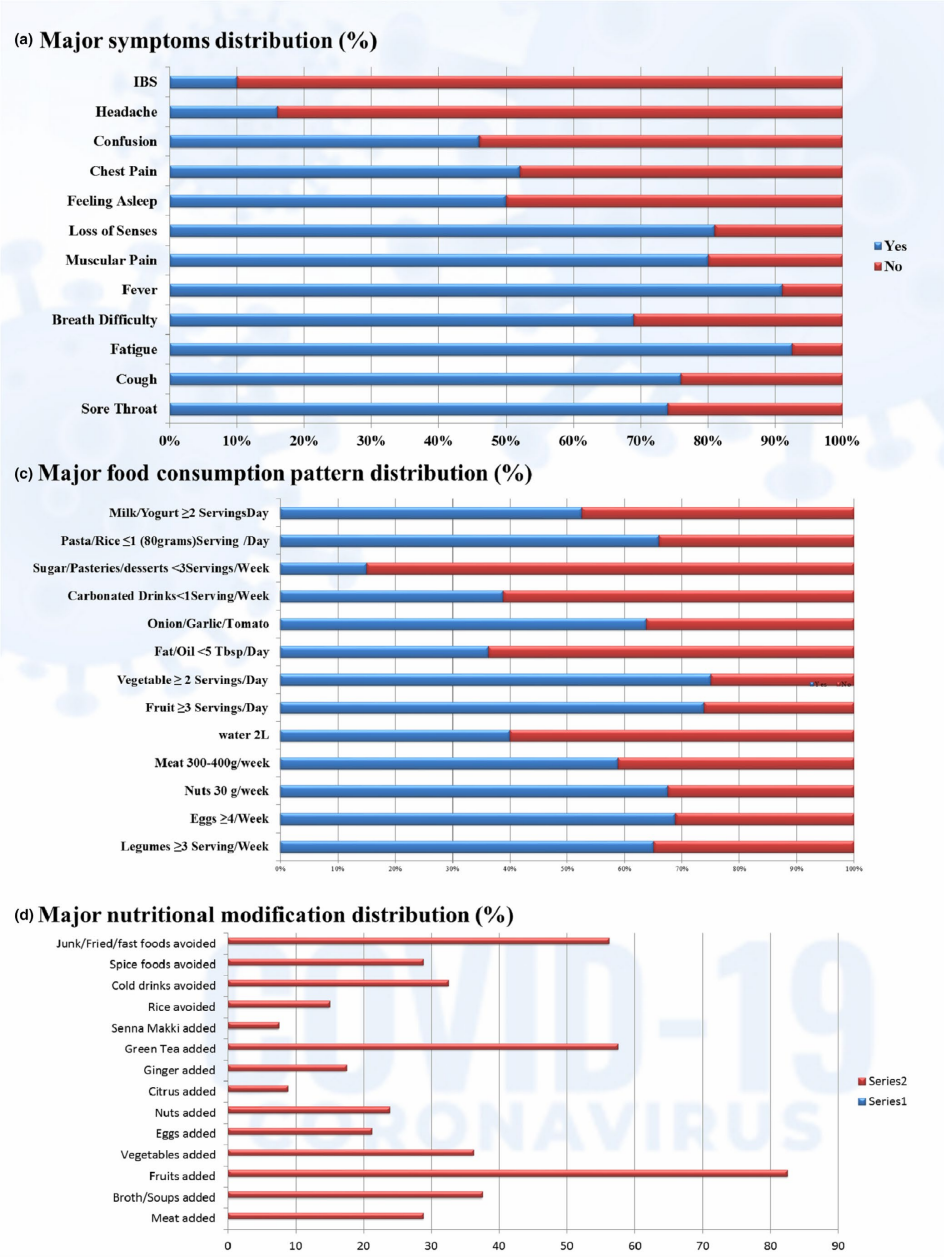
Symptoms, food consumption and nutritional modification distribution (%)

**FIGURE 5:**
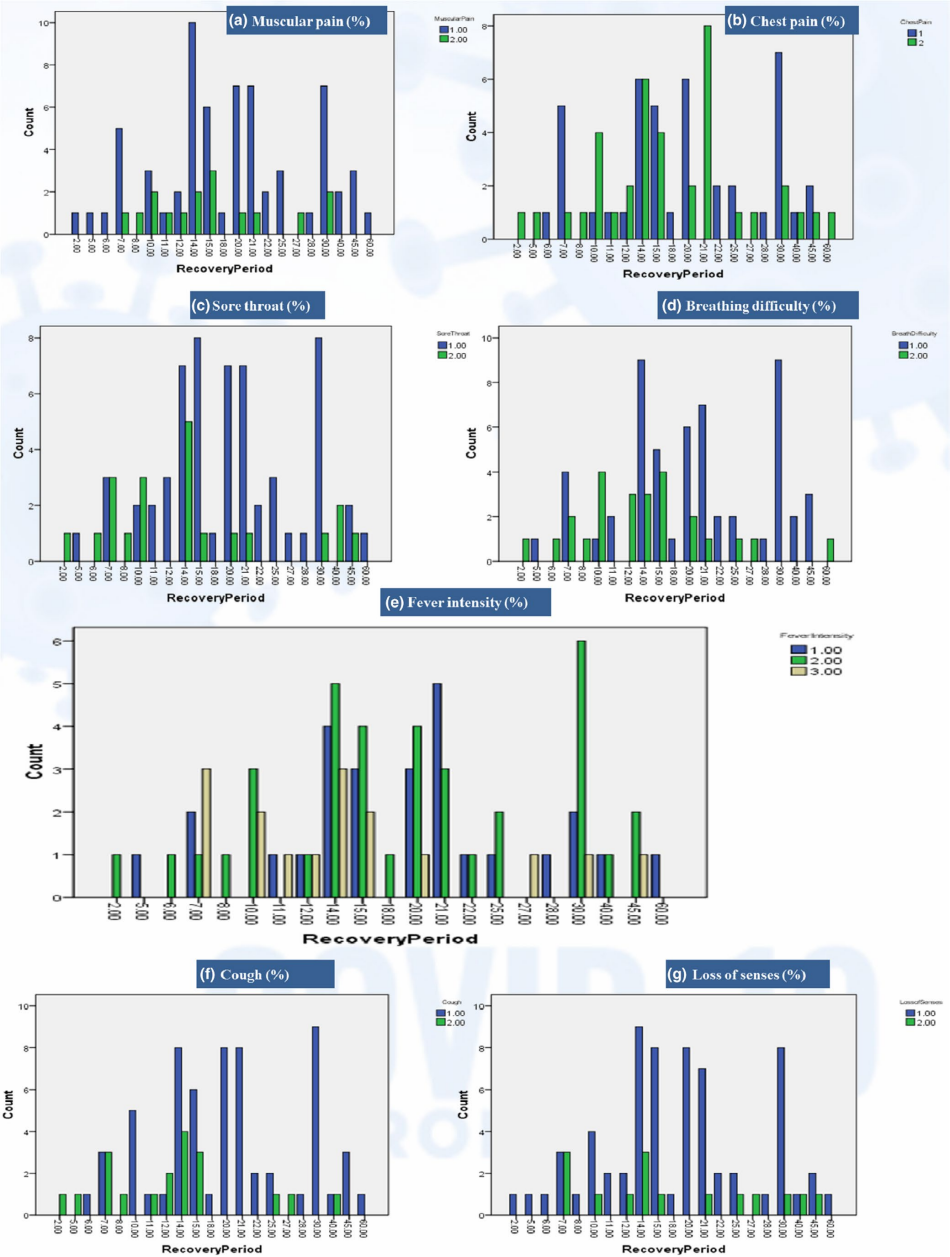
Apparent symptoms distribution along recovery period. ^*^a, b, c, d, f, g (1 = yes, 2 = no); e (fever intensity [1 = high, 2 = moderate, 3 = mild])

The percentage distribution of apparent symptoms is represented in Figure 4a. The most dominant symptoms (>80%) were fatigue, fever, loss of senses, and muscular pain, while a cough, sore throat, and breathing difficulty were fairly dominant (60%–80%). Symptoms like chest pain, drowsiness, and confusion were 40%–60% distributed, while headache and irritable bowel syndrome (IBS) were least distributed (<20%). The presence of a sore throat, cough, breathing difficulty, fever, fever consistency, fever intensity, drowsiness, and chest pain had significant associations and correlations with the recovery period, as shown in Table 3. Muscular pain, loss of senses, confusion, headache, and IBS were nonsignificant on recovery. The symptoms distribution along with recovery period in Figure 5 also shows the distribution of more consistent symptoms like sore throat, cough, breathing difficulty, fever, and fatigue in the delayed recovery zone, which supports their delaying effect beyond 2 weeks.

**TABLE 3:** Statistical associations and correlations of all variables in study data

	Variables		*χ* ^2^	LR	LA	Λ	GK	Γ	r_s_	r_p_
Personal data	Gender	Male	21.551	25.676	3.811	0.286	0.269	−0.315	−0.243	−0.220
		Female	0.365	0.177	0.051^*^	0.040^*^	0.381	0.032^*^	0.030^*^	0.050^*^
	Age	<40 years	7.874	317.105	6.925	0.284	0.266	0.223	0.31	0.296
	14−80 years	>40 years	0.419	1.000	0.008^***^	0.000^***^	0.150	0.001^***^	0.005^***^	0.008^***^
	BMI		1.275	366.503	0.043	0.219	0.251	0.057	0.075	0.023
			0.027^*^	1.000	0.836	0.000^***^	0.570	0.492	0.510	0.838
Drugs & supplements	Supplements	Multivitamin	19.331	24.233	0.751	0.273	0.242	−0.117	−0.093	−0.098
			0.500	0.232	0.386	0.044^*^	0.516	0.404	0.413	0.390
		Vitamin A	5.734	6.395	0.237	0000	0.072	0.112	0.033	0.055
			0.999	0.998	0.626	0000	0.999	0.527	0.772	0.629
		Vitamin D	23.783	27.189	1.348	0.211	0.297	0.261	0.178	0.131
			0.252	0.130	0.246	0.040^*^	0.266	0.116	0.114	0.248
		Vitamin C	22.859	27.926	0.418	0.382	0.286	0.037	0.03	0.073
			0.296	0.111	0.518	0.016^**^	0.310	0.798	0.794	0.522
		Vitamin E	42.865	19.455	3.088	0.500	0.536	−0.301	−0.109	−0.198
			0.002^***^	0.492	0.079	0.152	0.003^***^	0.271	0.338	0.079
		Zinc	24.934	28.395	0.188	0.333	0.312	−0.010	−0.007	−0.049
			0.204	0.100	0.665	0.028^*^	0.216	0.946	0.950	0.667
	Drugs	Analgesics & antipyretics	14.896	19.416	0.186	0.143	0.186	0.054	0.041	0.048
			0.782	0.495	0.666	0.282	0.793	0.712	0.718	0.669
		Antibiotics	14.091	18.719	0.002	0.237	0.176	0.005	0.004	0.005
Major food consumption pattern			0.826	0.540	0.966	0.194	0.835	0.969	0.969	0.966
	Water		77.143	74.922	3.113	0.256	0.264	−0.204	−0.198	−0.199
			0.570	0.640	0.078	0.005^***^	0.376	0.075	0.078	0.078
	Egg		1.012	86.939	1.465	0.156	0.238	−0.097	−0.193	−0.136
			0.893	0.990	0.226	0.156	0.670	0.405	0.362	0.228
	Nuts 30 gram/week		26.084	29.474	0.001	0.346	0.326	0.020	0.016	−0.003
			0.163	0.079	0.979	0.044^*^	0.174	0.893	0.890	0.979
	Fish		26.098	30.202	0.383	0.292	0.326	0.104	0.077	0.070
			0.163	0.067	0.536	0.065	0.173	0.475	0.497	0.539
	Meat 300−400 g/week		15.732	19.297	0.018	0.273	0.197	0.100	0.080	0.015
			0.733	0.503	0.894	0.055^*^	0.745	0.468	0.483	0.895
	Legumes ≥3 serving/Week		21.184	26.43	8.954	0.214	0.265	0.366	0.283	0.337
			0.386	0.152	0.003^***^	0.011^**^	0.402	0.009^***^	0.011^**^	0.002^***^
	Fruit ≥3 servings/Day		26.365	30.615	0.099	0.286	0.33	0.01	0.007	0.035
			0.154	0.060	0.753	0.052^*^	0.165	0.949	0.948	0.756
	Vegetable ≥2 servings/Day		13.007	14.655	0.106	0.100	0.163	0.051	0.036	0.037
			0.877	0.796	0.745	0.152	0.884	0.752	0.753	0.747
	Fat/Oil <5 Tbsp/Day		15.608	18.761	0.709	0.207	0.195	0.138	0.106	0.095
			0.741	0.537	0.400	0.128	0.752	0.330	0.348	0.403
	Onion/Garlic/Tomato		20.657	25.017	0.786	0.276	0.258	0.165	0.128	0.100
			0.418	0.201	0.375	0.082	0.433	0.260	0.259	0.379
	Carbonated drinks <1 serving/Week		14.776	18.573	0.438	0.161	0.185	0.139	0.109	0.074
			0.789	0.550	0.508	0.409	0.799	0.327	0.337	0.511
	Sugar/Pasteries/desserts <3 servings/Week		21.068	21.705	0.251	0.083	0.263	0.039	0.023	0.056
			0.393	0.357	0.616	0.645	0.409	0.838	0.841	0.619
	Pasta/Rice ≤1 (80grams) serving/Day		75.63	72.889	0.213	0.250	0.27	−0.041	−0.044	−0.052
			0.618	0.701	0.645	0.040^*^	0.322	0.721	0.700	0.648
	Milk/Yogurt ≥2 servings day		64.145	64.912	5.919	0.204	0.217	−0.294	−0.305	−0.274
			0.902	0.889	0.015^**^	0.044^*^	0.812	0.005^***^	0.006^***^	0.014^**^
Nutritional modifications (Foods added/avoided)	Diet improved		33.764	36.223	1.437	0.217	0.311	−0.154	−0.117	−0.135
			0.746	0.641	0.231	0.053^*^	0.152	0.290	0.303	0.233
	Meat added		24.226	29.101	1.241	0.261	0.303	0.224	0.163	0.125
			0.233	0.086	0.265	0.052^*^	0.246	0.164	0.149	0.268
	Broth/Soups added		18.406	23.655	0.814	0.200	0.230	0.122	0.095	0.102
			0.561	0.258	0.367	0.285	0.576	0.394	0.402	0.370
	Fruits added		13.949	14.914	0.083	0.071	0.174	0.012	0.007	−0.032
			0.833	0.781	0.773	0.314	0.842	0.945	0.950	0.775
	Vegetables added		22.327	29.230	0000	0.214	0.279	−0.084	−0.065	0.002
			0.323	0.083	0.984	0.173	0.338	0.547	0.567	0.985
	Eggs added		12.889	16.731	1.136	0000	0.161	0.134	0.086	0.120
			0.882	0.670	0.287	1.000	0.889	0.381	0.446	0.289
	Nuts added		17.985	21.000	0.004	0.158	0.225	−0.012	−0.008	−0.007
			0.588	0.397	0.950	0.175	0.603	0.943	0.942	0.951
	Citrus added		25.032	19.971	2.113	0.143	0.313	0.367	0.167	0.164
			0.200	0.460	0.146	0.563	0.212	0.207	0.138	0.147
	Ginger added		25.647	26.66	0.002	0.214	0.321	−0.082	−0.050	−0.005
			0.178	0.145	0.963	0.175	0.189	0.640	0.659	0.963
	Green tea added		14.845	19.586	0.897	0.176	0.186	−0.078	−0.062	−0.107
			0.785	0.484	0.344	0.152	0.796	0.577	0.584	0.347
	Rice avoided		15.403	18.056	0.385	0000	0.193	0.051	0.029	0.070
			0.753	0.584	0.535	1.000	0.764	0.770	0.799	0.538
	Cold drinks avoided		24.603	31.009	0.636	0.192	0.308	0.138	0.104	0.090
			0.217	0.055^*^	0.425	0.409	0.230	0.329	0.356	0.428
	Spice foods avoided		24.985	27.337	8.004	0.261	0.312	−0.347	−0.258	−0.318
			0.202	0.126	0.005^***^	0.103	0.214	0.03^*^	0.021^*^	0.004^***^
	Junk/Fried/fast foods avoided		16.192	19.45	3.496	0.286	0.202	−0.266	−0.213	−0.21
			0.705	0.493	0.062	0.062	0.717	0.048^*^	0.058^*^	0.061
Lifestyle	Smoking		19.987	16.339	0.031	0.143	0.250	0.068	0.031	0.020
			0.459	0.695	0.861	0.314	0.474	0.807	0.786	0.862
	Sleep hours		98.471	74.792	2.499	0.333	0.276	−0.179	−0.183	−0.178
			0.079	0.644	0.114	0.003**	0.271	0.118	0.105	0.114
	Physical activity before COVID		33.21	36.288	4.244	0.333	0.227	0.165	0.143	0.232
			0.768	0.638	0.039^*^	0.026^*^	0.654	0.219	0.207	0.039^*^
	Physical activity during COVID		34.548	37.857	1.568	0.308	0.240	0.176	0.147	0.141
			0.714	0.567	0.211	0.029^*^	0.566	0.195	0.194	0.213
	Weight change		48.448	52.641	0.127	0.341	0.300	−0.041	−0.034	−0.040
			0.169	0.087	0.721	0.005^***^	0.196	0.732	0.764	0.724
	Steam		19.402	23.158	3.811	0.214	0.243	0.309	0.237	0.220
			0.496	0.281	0.051^*^	0.235	0.512	0.027^*^	0.034^*^	0.050^*^
Stress	Anxiety/stress		15.125	19.433	0.908	0.111	0.189	−0.086	−0.066	−0.107
			0.769	0.494	0.341	0.077	0.780	0.559	0.563	0.344
	Fear of death		22.544	29.149	0.025	0.207	0.282	0.128	0.099	0.018
			0.312	0.085	0.875	0.152	0.326	0.353	0.382	0.876
	PSTD		16.893	17.342	0.017	0.071	0.211	−0.062	−0.038	−0.015
			0.660	0.631	0.896	0.739	0.674	0.722	0.738	0.897
Diseases	Lung disease		16.689	17.944	0.358	0.091	0.209	0.097	0.054	−0.067
			0.673	0.591	0.550	0.314	0.686	0.665	0.636	0.553
	Autoimmune disease		47.251	21.664	0.285	0.500	0.591	0.075	0.027	0.060
			0.001^***^	0.359	0.594	0.152	0.001^***^	0.768	0.809	0.597
	Chronic disease		19.043	17.126	0.990	0.125	0.238	−0.279	−0.136	−0.112
			0.519	0.645	0.320	0.563	0.535	0.229	0.229	0.323
	Diabetes mellitus		25.425	23.855	1.875	0.182	0.318	−0.361	−0.201	−0.154
			0.186	0.249	0.171	0.314	0.197	0.038^*^	0.073	0.172
Symptoms	Sore throat		29.321	32.323	1.827	0.286	0.367	−0.36	−0.258	−0.152
			0.082	0.040^*^	0.176	0.052^*^	0.089	0.036^*^	0.021^*^	0.178
	Cough		33.063	39.474	6.422	0.263	0.413	−0.483	−0.337	−0.285
			0.033^*^	0.006^***^	0.011^**^	0.053^*^	0.037^*^	0.002^***^	0.002^***^	0.010^**^
	Fatigue		33.554	22.273	0.747	0.333	0.419	−0.200	−0.085	−0.097
			0.029^*^	0.326	0.388	0.152	0.033^*^	0.350	0.455	0.391
	Breath difficulty		35.097	42.026	5.246	0.440	0.439	−0.456	−0.347	−0.258
			0.020^*^	0.003^***^	0.022^*^	0.001^***^	0.022^*^	0.001^***^	0.002^***^	0.021^*^
	Fever		16.578	13.943	3.078	0.143	0.207	−0.503	−0.226	−0.197
			0.680	0.833	0.079	0.314	0.693	0.026^*^	0.044^*^	0.079
	Fever consistency		22.987	28.568	2.197	0.41	0.287	−0.241	−0.195	−0.167
			0.289	0.097	0.138	0.005^***^	0.304	0.074	0.083	0.139
	Fever intensity		33.896	39.397	1.172	0.209	0.203	−0.165	−0.160	−0.122
			0.780	0.497	0.279	0.055^*^	0.808	0.156	0.156	0.282
	Muscular pain		16.424	17.474	1.68	0.125	0.205	−0.214	−0.137	−0.146
			0.690	0.622	0.195	0.152	0.703	0.197	0.225	0.197
	Loss of senses		16.31	17.578	0000	0.067	0.204	−0.078	−0.049	−0.002
			0.697	0.615	0.988	0.314	0.710	0.689	0.663	0.988
	Feeling asleep		21.089	27.099	1.028	0.400	0.264	−0.157	−0.126	−0.114
			0.392	0.133	0.313	0.012^**^	0.407	0.255	0.265	0.316
	Chest pain		28.226	35.757	0.283	0.447	0.353	−0.104	−0.084	−0.060
			0.104	0.016^**^	0.594	0.000^***^	0.112	0.451	0.451	0.457
	Confusion		17.202	22.932	0.289	0.270	0.215	−0.069	−0.055	−0.060
			0.640	0.292	0.591	0.062	0.654	0.623	0.628	0.594
	Headache		22.176	22.309	0.121	0.154	0.277	0.165	0.099	0.039
			0.331	0.324	0.728	0.314	0.346	0.412	0.383	0.730
	IBS		18.735	16.75	0.012	0.125	0.234	−0.189	−0.091	−0.012
			0.539	0.669	0.913	0.314	0.554	0.233	0.424	0.913

Abbreviations: GK, goodman and Kruskal tau; LA, linear by linear association; LR, likelihood ratio; rp, Pearson's R; rs, Spearman's correlation; Γ, gamma; λ, lambda; χ2, chi‐square.

Significance levels: ^NS^p<0.05 *p ≤ .05; **p ≤ .01; ***p ≤ .001.

### 3.4 Lifestyle changes

**FIGURE 6:**
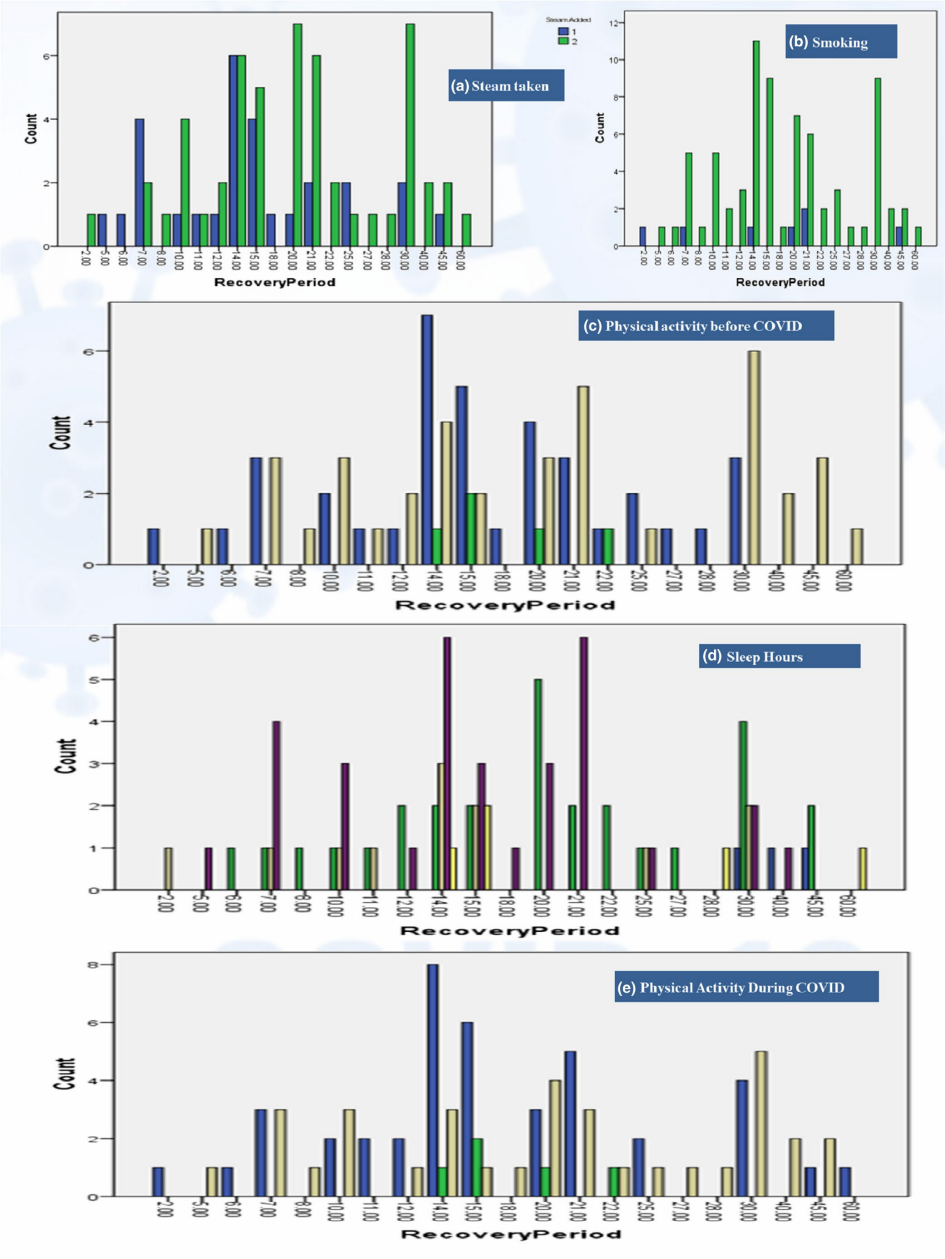
Life style (steam, smoking, sleep & activity) distribution along recovery period. ^*^a, b (1 = yes, 2 = no); c, e (1 = very active, 2 = fairly active, 3 = less/No active); d (5 = ≤5, 6 = 6, 7 = 7, 8 = 8, 9 = ≥9 hr/day)

The patients' responses for sleep hours were distributed from 3 to 10 hr with a mean of 7.1 ± 1.3 hr. The most frequently adopted lengths of sleep were 8 hr (40%), followed by 6 hr (35%), 7 hr (15%), ≥9 hr (6.2%), and ≤5 hr (3.8%). Statistical analysis revealed that length of sleep had a highly significant association with the recovery of patients (λ = 0.333, *p* = .003^***^). The sleep hour distribution (Figure 6d) revealed that 7 hr of sleep were less likely to be associated with late recovery. This association was strengthened by the results of a *t* test of comparison at <seven and ≥7 hr (t = −2.247, *p* = .027^*^). Only 8.8% of patients claimed that they smoked, and this had no significant association or correlation with the recovery period. There were 35% of patients reported to take steam inhalation daily during COVID‐19 infection, and statistical analysis showed a significant association with recovery (LA =3.811, *p* = .051^*^; Γ = 0.309, *p* = .027^*^; r_s_ = 0.237, *p* = .034^*^; r_p_ = 0.220, *p* = .050^*^). There were 66.2% of patients who faced anxiety/stress during quarantine, while 63.8% claimed to have fears including fear of death or disease transmission to others and being a cause of their death. Almost 17.5% claimed to face PSTD even after their complete recovery. Here, statistical analyses revealed no significant associations with recovery.

The results for physical activity before their COVID‐19 infection revealed that 46.2% of patients were active by doing regular walks, while only 6.2% had a very active status by doing gym/running/yoga/exercise. The remaining 47.5% were not engaged in any physical activity beyond their daily chores. During COVID‐19 infection quarantine, patients with an active status increased to 51% by motivating them to do indoor walks, while those who had a very active status remained persistent by changing the mode of their activities to weightless workouts/yoga. The remaining 42.5% were not engaged in any kind of physical activity during quarantine. Statistical analysis revealed a significant association of physical activity before COVID‐19 infection with recovery (LA =4.244, *p* = .039; λ = 0.333, *p* = .026^*^; r_s_ = 0.232, *p* = .039^*^). On further comparison between physically active and nonactive status with recovery, significant variances were reported (t = 2.153, *p* = .034^*^). Being physically active during COVID‐19 infection quarantine was also found to be significantly associated with the recovery period (λ = 0.308, *p* = .029^*^). It is clear from Figure 6 that less/no active patients are more confined to the late recovery zone, but very active status (vigorous exercise) during COVID‐19 infection can also negatively affect recovery.

### 3.5 Dietary modifications

#### 3.5.1 Major food consumption pattern followed

The major food consumption (%) pattern is shown in Figure 4b. The water intake of patients ranged from 0.75 to 2.5 L. Only 16.2% drank more than 2 L per day, while 23.8% drank about 2 L daily. The most common consumption volume was 1.5 L by 46.2%, and the least common consumption volume was 0.75 L by only 2.5%. The remaining 11.2% drank 1 L/day. Water consumption was significantly associated with recovery (λ = 0.256, *p* = .005^***^). Results were also significant (t = 3.006, *p* = .037^*^), when comparing for ≥2 L and <2 L. Meat consumption was <1 serving/day or 3–4 servings/week (one serving =100–150 g) by 58.8% of patients and showed significance on recovery (λ = 0.273, *p* = .055^*^). There were 65% of patients who reported having ≥3 servings (one serving =150 g) of legumes each week. Statistical analysis revealed highly significant associations and correlations of legume intake with recovery (LA =8.954, *p* = .003^***^; λ = 0.214, *p* = .011^**^; Γ = 0.366, *p* = .009^***^; r_s_ = 0.283, *p* = .011^**^; r_p_ = 0.337, *p* = .002^***^). Nuts (30 g/week) consumption was reported by 65.5% of patients, and this showed significant association with recovery (λ = 0.346, *p* = 0. 044^*^). About 74% of patients had ≥3 servings (one serving =80 g) of fruits, and this was significantly associated with recovery (λ = 0.286, *p* = .052^*^). The lower intake of pasta/rice at ≤1 serving/day (one serving =80 g) was reported in 66% of patients and was significantly associated with recovery (λ = 0.250, *p* = .040^*^). Milk/yogurt consumption at ≥2 servings/day (one serving =150 ml) was reported by 52.5% patients and was significantly associated with recovery (LA =5.919, *p* = .015^**^; λ = 0.204; *p* = .044^*^; Γ = −0.294, *p* = .005^**^; r_s_ = −0.305, *p* = .006^**^; r_p_ = −0.274, *p* = .014^**^). Here, negative correlations indicate an inverse correlation with milk/yogurt quantity, that is, long recovery is more associated with the small portions. Egg consumption varied from zero to seven per week, for which the most common was four eggs/week (43.8%) followed by two eggs (17.5%), six eggs (13.8%), and seven eggs (11.2%), while only 3.8% consumed egg once a week and 8.8% did not eat eggs. Fish intake at ≥3 servings/week (one serving =100–150 g) was consumed by 30% of patients, while the remaining 70% had lower or minimal intake. Vegetables at ≥2 servings/day (one serving =80 g) were consumed by 75% of patients. Surprisingly, egg, fish, and vegetable consumption remained nonsignificant throughout the recovery period (Table 3).

#### 3.5.2 Major dietary changes (foods added/avoided/meal changes)

Dietary changes (%) that were made during COVID‐19 infection include the addition or exclusion of some foods. Major additions were meat, broths/soups, fruits, vegetables, eggs, nuts, ginger, green tea, and citrus, while major exclusions were rice, cold drinks/water/cola, spicy, and junk/fast/fried foods, as shown in Figure 4c. Only the addition of meat showed significant dependency on recovery (λ = 0.261, *p* = .052^*^). The addition of more soups or broths, vegetables, nuts, and citrus showed slight dependencies that were nonsignificant. Green tea was taken by 57.5% of patients regularly during quarantine, and 17.5% claimed to add ginger in their tea, where ginger has a slight dependency on recovery, but it was not significant. Senna makki (*Senna alexandrina*) herb was adopted by only 7.5% of patients as a remedial use for which statistical results revealed no significant association with the recovery period.

Among the most avoided items were junk/fast/fried foods in 56.2% patients, and these had significant effects on recovery (Γ = −0.266, *p* = .048^*^; r_s_ = −0.213, *p* = .058^*^). The second most avoided category was cold water/sodas/carbonated drinks in 32% of patients, which showed significant effects on recovery (LR =31.009, *p* = .055^*^). Patients who avoided spicy foods (28.8%) were also found to be significant (LA =8.004, *p* = .005^***^; Γ = −0.347, *p* = .030^*^; r_s_ = −0.258, *p* = .021^*^; r_p_ = −0.318, *p* = .004^***^), while those who avoided rice (15%) were nonsignificant.

In the current study, 41.2% added a meal or snack, 20% skipped a meal, and 38.8% had no change in their diet as it was already considered to be healthy. The recovery period showed dependency toward changes in meals/snacks (λ = 0.383, *p* = .000^***^); therefore, meal management for achieving a healthy and balanced diet during infection was important for influencing the recovery. Overall, 71.2% of patients claimed that they had improved their dietary patterns during COVID‐19. Statistical analysis revealed that this positive attitude about their improvement in diet also influenced the recovery period significantly (λ = 0.217, *p* = .053^*^).

#### 3.5.3 Major nutritional supplements and drugs taken

**FIGURE 7:**
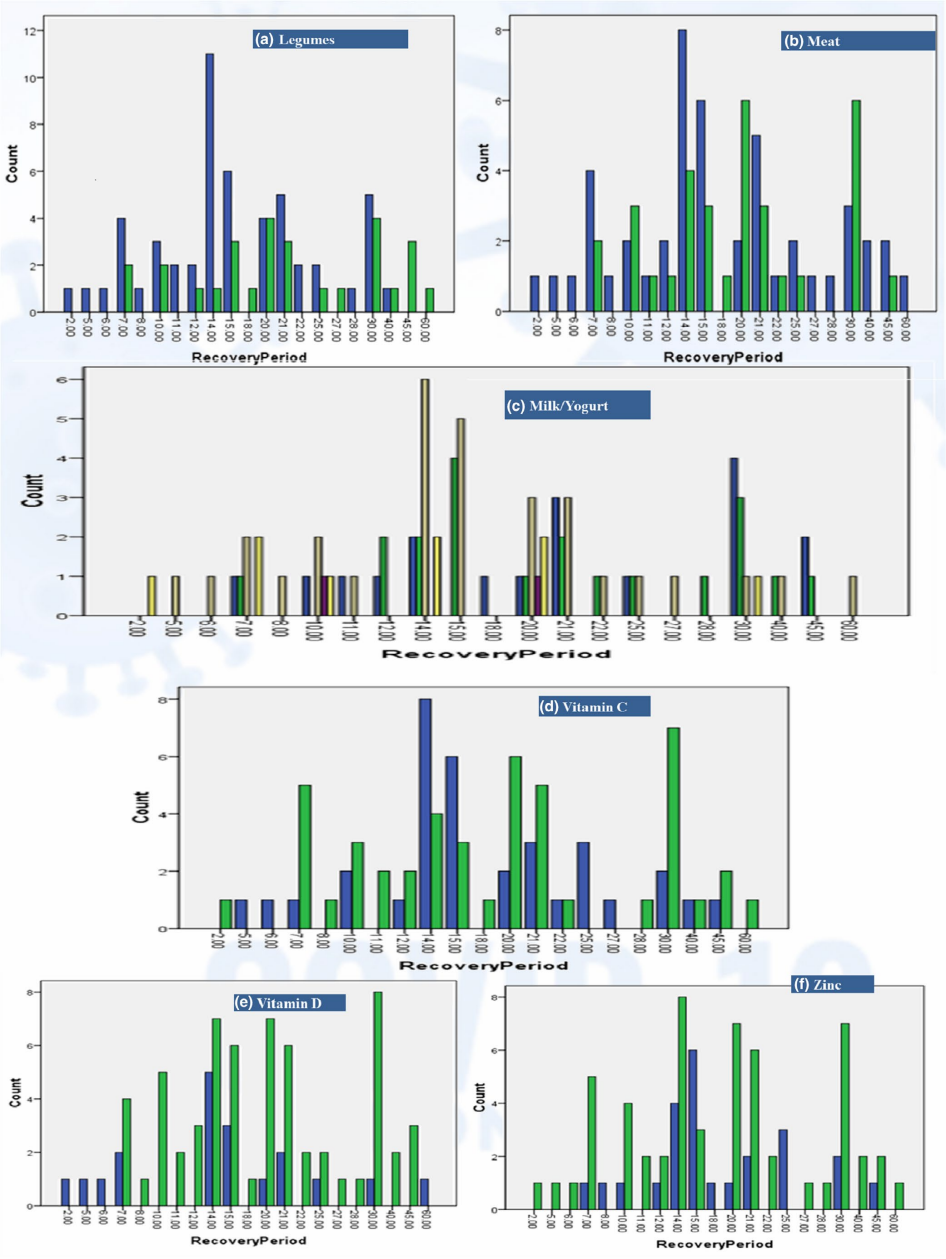
Major food and supplements consumption distribution along recovery period. *a, b, d, e, f (1 = yes, 2 = no); c (0.5 to 3 portions, where 1 portion =150 ml)

To meet additional nutritional requirements and to fulfill any existing deficiencies, 41.2% of patients took daily supplemental doses of multivitamins that were found to be significant on recovery (λ = 0.273, *p* = .044^*^). Here, 42.5% added a vitamin C supplement that was also significant on recovery (λ = 0.382, *p* = .016^**^). Zinc was taken daily by 30% and was found to be significant (λ = 0.333, *p* = .028^*^), and vitamin D (23.8%) also showed significant results (λ = 0.211, *p* = .04^*^). Only 5% of patients took vitamin E supplements for which the results showed high significance (*χ* ^2^ = 42.865, *p* = .002^***^; GK =0.536, *p* = .003^***^). Vitamin A (3.8%) was nonsignificant (Figure 7).

Among the most used type of drugs were antipyretic analgesics (Panadol =86%, Nims =7.6%, and acetaminophen =6%) taken by 65% of patients, while the second most used type of drugs were antibiotics (47%) (Azomax/Azithromycin =92%), but the results for the drugs used in this study were not significant.

## 4 DISCUSSION

COVID‐19 infected, home quarantined patients from Punjab, Pakistan, recovered from this infection in a mean time of 2.8 weeks or 19 days. Although the recovery period has slight variations owing to seasonal and environmental changes, an almost similar recovery period of 21 days has been reported in a study from Barman et al. (2020) and there was a slight increased recovery period of 25 days in India during March to April 2020 (Barman et al., 2020).

Among gender distribution in COVID‐19 infected patients, males were greater in number. The higher incidence of COVID‐19 infection in males could be due to religious, cultural, and social civilization patterns of Pakistani nationals, where males dominantly move around for earning purposes as compared to the females who are mostly confined to home chores. Moreover, females are generally more resistant to infections than males, and this may be influenced by several factors, including sex hormones and high expression of coronavirus receptors (ACE 2) in men, along with other lifestyle factors (Bwire, 2020). Not only were men more exposed to COVID‐19 infection, but also the results for a delayed recovery period were also more distributed among men as compared to women (Figure 2d). These results are in agreement with other published works that correlate males with a higher severity, morbidity, and mortality due to COVID‐19 (Griffith et al., 2020; Peckham et al., 2020; Pradhan & Olsson, 2020). The whole scenario of females having an advantage against COVID‐19 could be due to the prevalence of gender variations in both innate and adaptive immune systems. Especially in the adaptive immune system, where females have more CD4+ T cells, more robust CD8+ T‐cell cytotoxic activity and improved B‐cell immunoglobulin output than males (Peckham et al., 2020).

The results on age distribution showed significant associations with recovery, which ultimately concluded delayed recoveries in older age (Figure 2c). This was especially significant after the age of forty, and this outcome is supported by other studies claiming that in comparison with younger patients, elderly patients are more vulnerable to COVID‐19 infection and have a worse outcome, primarily due to decreased or compromised immune functioning (Chowdhury et al., 2020; Silverio et al., 2020; Verity et al., 2020). Other factors contributing to this recovery delay in older age could be emotional distress, fears associated with isolation, health vulnerabilities, comorbidities, and dependency (Morrow‐Howell et al., 2020; Shahid et al., 2020).

In our results, BMI association was also found to be significant on the recovery period and this is supported by Silverio et al. (2020) who claimed that obesity is widespread among COVID‐19 infected hospitalized patients. Obesity can be a cause of impaired immune response and delayed recovery and hence can serve as an independent risk factor for the severity of COVID‐19 pathogenesis (Morais et al., 2020). Reasons behind this association could be the link of obesity with lower expiratory volume and functional capability of the respiratory system. Especially in patients with elevated abdominal obesity, reduced diaphragmatic excursion can compromise pulmonary functions. Moreover, increased inflammatory cytokines linked to obesity can also play a role in the rise of the negative prognosis, which could ultimately delay recovery (Cena & Chieppa, 2020; Dietz & Santos‐Burgoa, 2020; Popkin et al., 2020). Not only does increased weight has negative outcomes associated with recovery, but also unnecessary weight loss during the infection period was also found to be more prevalent in patients with late recoveries (Figure 3b). This unplanned weight loss could be due to a disturbing hunger pattern, nausea, or diarrhea due to IBS and/or higher energy and protein utilization by the body to fight infection. Not eating properly could result in malnutrition (Anker et al., 2021; Filippo et al., 2020), which can impair the immune strength and ultimately delay recovery.

As far as the results for previous medical history were concerned, the prevalence of autoimmune disorders and diabetes mellitus imparted significant delays in recovery, as these can impair the immune system and ultimately serve as a risk factor for delayed recovery from COVID‐19 (Alagawany et al., 2021; Liu & Liu, 2020). Among the results for apparent symptoms, the presence of a sore throat, cough, breathing difficulty, fever, and chest pain were associated with delayed recovery. The results are in agreement with previous studies of Alagawany et al. (2021) and Liu and Liu (2020) for symptoms of fever (83%–98.6%) and cough (46%–82%) and a lower presence of other mild symptoms such as chest pain, headache, breathing difficulty, and body pains.

Among the lifestyle activities, sleep hours were significant on COVID‐19 recovery, especially sleep of <7 hr was distributed more among patients with late recoveries as indicated in Figure 6d and this is in agreement with Nizami and Uddin (2020). Sleep has been linked to immunity, whereby a deficiency of sleep impairs immune responses by downregulating immunological markers and their cells, and hence can impart a significant effect on COVID‐19 infection severity and recovery (de Sousa Martins e Silva et al., 2020; Liu et al., 2020). Smoking was found to be nonsignificant on the recovery period in our results, which agrees with the study by Rossato et al. (2020), but is in contrast to the study by Reddy et al. (2021). While comparing a number of studies, the effect of smoking on COVID‐19 infection has been identified as controversial (Polverino, 2020). In our study, the use of steam inhalation in COVID‐19 infection was found to be significant, which might be due to the thermal killing imposed by steam that reduces the viral load (la Marca et al., 2021; Swain & Sahu, 2021). The results for higher anxiety and fear prevalence among the patients are in agreement with those of Ettman et al. (2020) who states that there is higher depression and anxiety prevalence due to the COVID‐19 pandemic among the general population. A physically active status before and during COVID‐19 infection has revealed significant associations with the recovery period. Staying physically active along with good nutrition, weight management, and a stress‐free mind is crucial for a healthy immune system to combat infectious diseases like COVID‐19 (Khoramipour et al., 2021; Lange & Nakamura, 2020). Similar outcomes have been reported in a study on COVID‐19 infected healthcare workers engaged in a healthy diet and exercise (Do et al., 2020).

Results on major food consumption patterns revealed significant associations of water, legumes, nuts, meat, and fruit consumption with COVID‐19 recovery. The benefits of increased water consumption at ≥2 L in COVID‐19 have also been supported by de Faria Coelho‐Ravagnani et al. (2020). Water along with good nutrition plays a key role in the body's immune defense from COVID‐19 recovery (ASPEN, 2020). Highly significant results for legumes in this study could be due to the fact that these are rich sources of dietary fiber that also serve as prebiotics and B vitamins (Calder, 2020). Another reason could be the presence of daidzein and genistein in legumes that are predicted to be bioactive compounds for COVID‐19 treatment (Brahmaiah & Ankit, 2020). Similar is the case for nuts consumption that are rich in anti‐inflammatory omega‐3 polyunsaturated fatty acids, antioxidative and immune‐supporting minerals like zinc and selenium, and bioactive peptides that could be a reason behind their significant association with recovery (Kieliszek & Lipinski, 2020; Zabetakis et al., 2020). Fruits being rich in vitamins, minerals, and antioxidants were also significant during the recovery period. Riboflavin and beta‐carotene in fruits are especially among proven bioactive compounds against COVID‐19 (Brahmaiah & Ankit, 2020). A decrease in milk and/or yogurt consumption revealed a highly significant increase in the recovery period based on their inverse/negative correlations. The reason behind this apparent protection could be the rich riboflavin content in milk that is a bioactive compound against COVID‐19 (Brahmaiah & Ankit, 2020) and the rich microbiotics in yogurt that help in enhancing immunity (Antunes et al., 2020; Dhar & Mohanty, 2020). Among the results for major dietary changes in the study, the addition of meat was found significant, while all other remedial measures of taking green tea with or without ginger or taking senna makki (*Senna alexandrina*) herb, or eating garlic were nonsignificant. The avoidance of unhealthy dietary patterns including consumption of fast/fried/junk/spicy foods and cold drinks/sodas were found to be significant steps toward fast recovery. The use of vitamins C, D, E, and zinc as additional daily supplemental doses was found to be significant and was supported and suggested in COVID‐19 patients by various studies (Cena & Chieppa, 2020; Chowdhury et al., 2020; de Faria Coelho‐Ravagnani et al., 2020; Fernández‐Quintela et al., 2020; Iddir et al., 2020; Jayawardena et al., 2020) for the nutritional management of the disease. Other studies have supported the intake of vitamins and minerals, especially vitamin D, which was found to be deficient in COVID‐19 patients (Im et al., 2020; Kohlmeier, 2020). Similar dietary patterns have been recommended by the American Society for Parenteral and Enteral Nutrition (ASPEN, 2020). However, the use of supplements for the general public should be encouraged by seeking advice from a dietitian or physician to avoid adverse food–drug, drug–drug, or drug–medical treatment interactions. Overall, these dietary changes adopted by patients are a productive turn toward healthy lifestyle adherence and indicate that the general population has tried to focus on dietary improvements as guided by the WHO and FAO worldwide (FAO, 2020). Because nutritional status and diet can influence COVID‐19 outcome by modulating inflammation and immune function, dietary changes may be necessary (Silverio et al., 2020).

## 5 LIMITATIONS AND STRENGTHS OF THE STUDY

Limitations of the current study were a relatively small sample size because of a limited number of patient readily participating, difficulties in getting access to the positively detected, healthy recovered COVID‐19 patients, collecting all of the data on lengthy telephone calls, and limited resources due to no funding or supporting authorities. Similarly, the whole data reported by patients on anthropometrics, apparent symptoms, and past medical history were based on a patient's true knowledge. On the other hand, the strength of this study is its uniqueness as this type of cross‐sectional study was not conducted before in Punjab and/or Pakistan during the COVID‐19 pandemic according to current knowledge. There are plans to expand the study to larger sample size and geographical area using electronic online survey methodology and proper funding.

## 6 CONCLUSION

Timely nutritional and lifestyle changes can help us save our future generations. Based on this study, we have concluded many beneficial nutritional guidelines by examining the nutritional patterns adopted by COVID‐19 recovered home quarantined patients. A healthy lifestyle with appropriate sleep hours, steam inhalation, and physical activity could help fight COVID‐19 infection more effectively and/or more quickly. Similarly, increased water consumption, along with more intakes from plant‐based organic home‐cooked foods, while omitting nonhealthy junk and fast foods also facilitate early recovery. Taking a daily supplemental dose of selected vitamins (C, D, E), minerals (zinc), or taking them in combination as a daily multivitamin and mineral dose can enhance the body's ability to fight the infection and could facilitate early recovery. The outcomes of this study are very comprehensive and have set down a nutritional and lifestyle base for health promotion, early recovery, and a positive survival rate against the COVID‐19 pandemic. Considering all the above positive outcomes, we can modify our lifestyle to have a safe journey toward a future free of COVID‐19 generated fears.

## CONFLICT OF INTEREST

The authors report no conflicts of interest.

## APPENDIX 1

### Nutrition and life style modification questionnaire for COVID‐19 patients

**Table:**

Sr. No.	Questions	Answers
**I**	**Personal data**	
1	Age	
2	Gender	Female/male
3	Hometown/Province	City/Province
4	Current employment	Unemployed/Retiree/Student/I work in smart working at home/I go to the work as usual/I have currently suspended my job
**II**	**Anthropometrics**	
5	Weight in Kg	
6	Height in ft/cm	
7	BMI	
8	Body weight change	Gain/Loss/Stable
**III**	**COVID 19 update**	
9	COVID−19 Case Detection type:	(PCR confirmed, Antibody test confirmed, X‐ray/CT Scan confirmed, Physician suspected)
10	Was there someone in your house/family/friend tested positive for COVID 19 in the past month?	Yes/No
11	Had you travelled in the past 14 days to any regions affected by COVID 19?	Yes/No
12	Any idea from where did you get infected?	Hospital/Home/Job/Educational Institute/Festival/No Idea
**IV**	**Apparent symptom**	
13	Did you feel a sore throat?	Yes/No
14	Did you feel cough?	Yes/No
15	Did you feel fatigue?	Yes/No
16	Did you feel short of breath or difficulty breathing?	Yes/No
17	Did you have fever?	Yes/No
18	Did you have fever constantly?	Yes/No
19	What was the nature of fever?	Mild/Moderate/High
20	Did you have muscular pain?	Yes/No
21	Had you lost taste or smell scene?	Yes/No
22	Were you falling asleep frequently?	Yes/No
23	Was there any chest pain?	Yes/No
24	Did you feel confusion state of mind?	Yes/No
25	Did you feel nausea, diarrhea or irritable bowel syndrome (IBS)?	Yes/No
26	Do you have headache?	Yes/No
**V**	**Past medical history**	
27	Did you have asthma or any lung disorder?	Yes/No
28	Did you have any chronic diseases? If yes please mention?	Yes/No (High blood pressure/Heart disease/liver disease/kidney disease/Any other)
29	Do you have any autoimmune disease?	Yes/No
30	Were you diabetic?	Yes/No
**VI**	**Nutritional modifications**	
31	What food you avoided during COVID?	Rice/Fast/Fried/Junk/Spicy/Bakery/Cold/Carbonated Drinks/Citrus/Any other
32	What food you ate frequently during COVID?	Fruits/Vegetables/Nuts/Fish/Red Meat/Chicken/Eggs/Soups/Broths/Any other
33	Did corona bring back healthy habits?	Yes/No
34	What was Corona`s impact on food habits?	Positive/Negative
35	What remedies you used?	Steam/Warm Water/G. Tea/Ginger Tea/Garlic/Onions/Any other
36	Are ≤4 tablespoons of fat/oil used each day? If no then how much?	Yes/No
37	Are ≥2 servings (of 200 g each) of vegetables eaten each day? If no then how much?	Yes/No
38	Are ≥3 servings of fruit (of 80 g each) eaten each day? If no then how much	Yes/No
39	Is <1 serving (100–150 g) of meat products eaten each day? If no then how much	Yes/No
40	Is <1 serving (330 ml) of sweet or sugar sweetened carbonated beverages consumed each day?	Yes/No
41	Are ≥3 servings (of 150 g) of legumes consumed each week?	Yes/No
42	Are ≥2 servings of fish (100–150 g) or seafood (200 g) eaten each week?	Yes/No
43	Is <3 servings of commercial sweets/pastries eaten each week?	Yes/No
44	Is ≥1 serving (of 30 g) of nuts consumed each week?	Yes/No
45	Are pasta, vegetable or rice dishes flavored with garlic, tomato, leek or onion eaten ≥twice a week?	Yes/No
46	How many portions of pasta, rice or other cereals? (1 medium portion =80 g)	None/Half portions/1 portion/2 portions/> 2 portions
47	How many portions of milk or yogurt do you consume per day? (1 serving =150 ml in a cup or 125 g a jar)	None/Half portions/1 portion/2 portions/> 2 portions
48	How many portions of cheese or dairy products do you consume per week? (1 portion of dairy product =100 g; 1 portion of matured cheese =50 g)	None/Half portions/1 portion/2 portions/> 2 portions
49	How many eggs do you consume per week?	None/1 egg/2 eggs/4 eggs/> 4 eggs
50	Did your lifestyle and eating habits changed during the COVID−19 pandemic period?	No, they didn't/yes, it get worse/yes, it improved
51	Did you change the number of daily meals, during this period?	No, it did't/Yes, I skip 1 or more of the main meals (breakfast, lunch, dinner)/Yes, I skip 1 or more of snacks between meals/Yes I added 1 or more of the main meals/Yes, I added 1 or more of the snacks between meals/Yes, I eat out of the meals
52	How much water do you drink per day?	<1 L/1 L–2 L/> 2 L
**VII**	**Use of nutritional supplements and drugs**	
53	Did you take any multivitamin? If Yes please mention.	Yes/No
54	Did you take vitamin D supplements?	Yes/No
55	Did You take vitamin C supplement?	Yes/No
56	Did you take vitamin E supplements?	Yes/No
57	Did you take Zinc supplement?	Yes/No
58	Did you take any analgesic or antipyretic drug? If Yes please mention.	Yes/No
59	Did you take antibiotics? If yes please mention.	Yes/No
**VIII**	**Lifestyle modifications**	
60	Did you smoke before COVID−19 pandemic period? (cigarettes, cigarillos, cigars, electronic cigarette)	No/Yes
61	How many hours did you sleep?	<5 hr/5–7 hr/7–9 hr/> 9 hr per night
62	Did you walk or play sport before and after the COVID−19?	No/walk/gym/run/swimming/soccer/volleyball/basket/crossfit/dance/yoga/aerobic fitness/martial arts/tennis/aerial gymnastics/other
63	What activity you followed at quarantine?	No/walk/weightless workout/weight training at home/tapis roulant/functional training/yoga/postural gymnastics/other
64	Did you inhale steam regularly during infection.	Yes/No
65	Did you regularly check your BP, Glucose, and Oxygen level?	Yes/No
**IX**	**Recovery period**	
66	For how many days did you isolate yourself?	Yes/No
67	What was your average recovery time?	<2, 2,3,4,5,6, >6
**X**	**PSTD**	
68	Did you feel anxiety/stress during infection?	Yes/No
69	Did You feel any fear of following?	Fear of death/Fear of disease transmission
70	Are you still suffering from any post‐traumatic stress disorder?	Yes/No

Please attach your report for any of following (PCR test/Antibody test/CT Scan/X‐ray or physician prescription).
